# Phenolic and Theobromine Contents of Commercial Dark, Milk and White Chocolates on the Malaysian Market

**DOI:** 10.3390/molecules14010200

**Published:** 2009-01-05

**Authors:** Cheng Chia Meng, Abbe Maleyki Mhd Jalil, Amin Ismail

**Affiliations:** Department of Nutrition and Dietetics, Faculty of Medicine and Health Sciences, Universiti Putra Malaysia 43400, UPM Serdang, Selangor Darul Ehsan, Malaysia; E-mails: chiaming_peace@yahoo.com (C-C. M.), maleyki@yahoo.com (A-M. J.)

**Keywords:** Chocolates, Catechin, Epicatechin, Theobromine

## Abstract

Chocolate contains a wide range of antioxidants that includes soluble phenolic compounds (phenolic acids, catechin, epicatechin, and proanthocyanidins), insoluble polymeric phenolics and methylxanthines. The objective of this study was to determine phenolic and theobromine contents in dark (DC), milk (MC), and white (WC) chocolates commonly found in the Malaysian marketplace. Total phenolic and flavonoids were determined by means of a spectrometric assay, while catechin, epicatechin and theobromine were quantified using a reverse-phase HPLC method. Dark chocolates exhibited the highest phenolics and flavonoids contents, followed by milk and white chocolates. Catechin and epicatechin were major flavonoids detected in dark chocolates. Theobromine was detected in dark and milk chocolates, but not in white chocolates. A high correlation (r = 0.93) between total phenolic and flavonoid contents, indicating that the major phenolic compounds in dark chocolates belong to the flavonoid class. When nutrition and health promotion are of concern, dark chocolates would be recommended over milk and white chocolates owing to their higher contents of antioxidant phenolic compounds.

## Introduction

The antioxidant properties of cocoa polyphenols have generated great interest in their potential health benefits. Epidemiological studies have shown that polyphenols may help to reduce the risk of cardiovascular diseases [1,2]. Previous *in-vivo* studies revealed that cocoa products may have the potential to decrease the risk of degenerative diseases [3,4,5,6]. A meta-analysis of randomized controlled trials showed that the intake of cocoa-rich foods may reduce blood pressure [7]. Recently, an on-going cohort study of the Moli-sani Project revealed that regular consumption of small amounts dark chocolate may have helped reduce serum C-reactive protein levels in healthy Italian subjects [8]. 

Cocoa contains a wide range of antioxidants, which includes soluble phenolic compounds and insoluble polymeric phenolics [9]. Cocoa bean is one of best known sources of dietary polyphenols, which comprise on average 12-18% of total weight on a dry basis [10]. Generally, cocoa contains significant amount of procyanidin monomers, namely catechin, epicatechin and dimer to tetradecamer [11,12,13,14,15,16]. 

Methylxanthines, such as caffeine, theobromine, and theophylline, are consumed daily in a variety of foods, beverages, and pharmaceutical products. Generally, theobromine is a caffeine metabolite [17]. It had been noticed that its major dietary sources are from chocolate foods and beverages [18]. Caffeine and theobromine are psychoactive compounds, but the pharmacologically active constituents responsible for certain mood changes and related effects of chocolates have not been tested [19]. Several studies have been reported on the theobromine content of some chocolate products, for example, hot chocolate [20,21], chocolate milk [20,22] and cocoa powder [20,23]. Limited information on methylxanthine contents of Malaysian manufactured chocolates has been published [24,25]. 

Chocolates may have different percentages of cocoa liquor, cocoa powder, cocoa butter, sugar, and milk powder used in making the different types of chocolates, namely dark, milk and white chocolates. The content of polyphenols and methylxanthines can vary depending on the source of beans (growing conditions and cocoa variety), processing conditions during fermentation and drying, and the chocolate making process. Alkalization (or dutching) of cocoa powder will influence the polyphenol [14,26] and methylxanthine contents [23]. Due to these factors, the ratio and types of these components found in cocoa beans are unlikely to be the same as those found in the finished products [27]. 

Bittersweet chocolates, or so-called dark chocolates, contain at least 15% cocoa liquor, but may contain as much as 60%, with the remainder being cocoa butter, sugar and other additives. Milk chocolates are the predominant form of chocolates consumed worldwide and typically contain 10-12% cocoa liquor. In Malaysia, many different brands of the various chocolate types are available; however, no report has been published on phenolic and theobromine contents of these chocolates. More research is needed on phenolic and methylxanhine analysis of chocolate from different brands, varieties or matrices which can be used to include or update food composition databases. Therefore, the present study aimed to investigate the amount of phenolic and flavonoid contents includes catechin and epicatechin and theobromine content in dark, milk and white chocolates available in the local Malaysian marketplace. 

## Results and Discussion

### Total phenolic and flavonoid contents

Table 1 shows total phenolic content in different types of commercial chocolates. The total phenolic content in chocolates was in the range of 116-585 mg catechin equivalent (CAE)/100 g. There was a significant difference in total phenolic content among different types of commercial chocolates (p < 0.05) as assessed by one-way ANOVA. The results obtained were in agreement with the study done by Grassi *et al*. [28], where 100 g of chocolate contained approximately 500 mg of polyphenols. Dark chocolate exhibited the highest phenolic content followed by milk and white chocolates. According to Cooper *et al*. [29], the present of non-fat cocoa solid (NFCS) as an excellent marker to determine the present of total phenolic content. Normally NFCS was found at the highest concentration in dark chocolates. Thus, theoretically, the higher amount of NFCS indicated the higher phenolic content in the chocolates. Nevertheless, white chocolates contained cocoa butter with no cocoa liquor. Thus, it cannot be considered as true chocolate. Hence, phenolic content and its compounds in white chocolates are lower than other chocolates. 

**Table 1:** Total phenolic and flavonoid contents in different types of commercial chocolates.

Type of chocolate	Total phenolic content (mg CAE/100 g chocolate)	Total flavonoid content (mg CAE /100 g chocolate)
Dark	578.64 ± 5.04	28.30 ± 1.92
Milk	160.46 ± 6.58	13.48 ± 1.54
White	126.39 ± 7.86	7.70 ± 0.55

Values are expressed as mean ± SD. All values are significant (p < 0.05) different between samples. The relative standard deviation was less than 11%.

The flavonoid content was in the range of 7–29 mg CAE/100 g chocolate (Table 1). This amount was lower when compared to the results reported by Grassi *et al*. [28]. The total phenolic and flavonoid contents of milk chocolates were significantly lower than for dark chocolates. Natsume *et al*. [16] reported that the presence of milk in cocoa products may interfere with analyses for polyphenols due to the binding of the latter to proteins. Almost all of chocolates available in the market contained added milk. Generally, dark chocolates contain either no or small amounts of milk, while white chocolates had added milk powder or condensed milk. 

**Figure 1:**
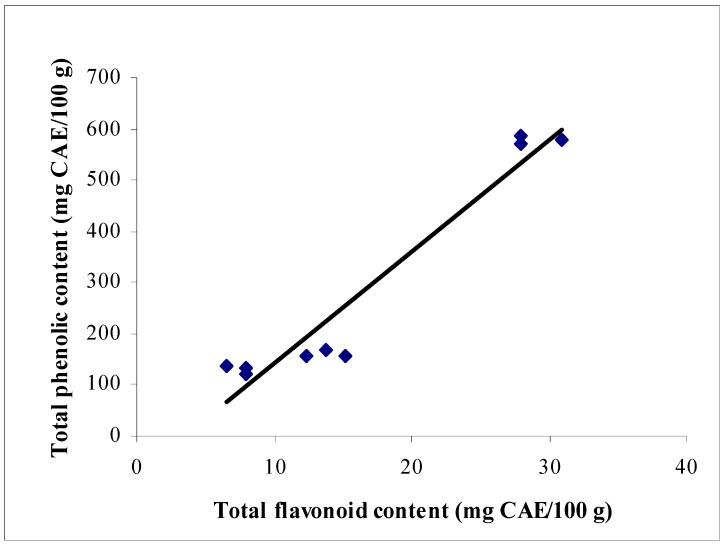
The correlation between total phenolic and flavonoid contents.

The flavonoid content was in the same order as the phenolic content. The content was significantly different (p < 0.05) among the studied commercial chocolates. A high correlation (r = 0.97) between total phenolic and flavonoid contents was observed (Figure 1). This may be attributed to catechin and epicatechin that are abundantly found in cocoa. According to Cooper *et al.* [29], epicatachin showed a strong correlation with other polyphenols, but the correlation between polyphenols and catechin was not high. The relationship suggests that whenever cocoa beans are processed into chocolates, these polyphenols were all affected to the same degree. 

The polyphenol content in chocolates varies greatly depending on processing techniques such as fermentation of cocoa beans and alkalinization of cocoa powders [30,14]. In addition, Cooper *et al*. [29] reported that the percentage of cocoa appears on the chocolates labeling cannot be used accurately to estimate the polyphenols concentration, since it includes polyphenol-free cocoa butter. This may overestimate the polyphenols concentration in particular chocolates. In fact, dietary polyphenols contain unlimited structure differences of carbon skeleton and oxidation state of the heterocyclic of flavonoids. This creates the difficulties in determination of polyphenols content. 

Fermentation is an essential step for the development of suitable flavours and flavour precursors that last from five to six days. However, epicatechin and soluble polyphenol content are reduced to approximately 10 to 20%, respectively, during fermentation [30]. This is not only due to oxidation processes, but is also caused by diffusion of polyphenols by sweating in fermentation [10,31,32]. Most beans for chocolate manufacture are fermented. Fermentation is one of the major steps that affect polyphenols content. Ecuadorian beans are normally fermented for three days and West African beans for five days. Between days two and three, epicatechin content is reported to decrease sharply, which could indicate that it is either consumed during the formation of larger tannins or lost due to water draining during fermentation [10]. 

According to Gu *et al*. [26], treatment with alkali influences the degradation of polyphenol compounds. Furthermore, Miller *et al.* [33] showed that alkalization reduced the antioxidant properties and flavonol content in cocoa powder. Most chocolates are produced using the alkalized cocoa powder, thus the content of polyphenols in chocolates had significant lower than in cocoa powder. 

### Catechin and epicatechin contents

Cocoa is reported to have high levels of antioxidant phenolics compared to tea [34]. Reversed-phase high performance liquid chromatography (HPLC) was used to determine the concentration of catechin and epicatechin in commercial chocolate samples. The spiking procedure with a known amount of standard (catechin and epicatechin) was done to confirm the identity of individual peaks. The limit of detection (LOD) and limit of quantification (LOQ) for catechin and epicatechin were 0.01 and 0.03 mg/mL, respectively. Catechin and epicatechin were detected in dark chocolate with 184.80 ± 1.14 and 274.35 ± 1.40 mg/100 g chocolate, respectively. These values were in agreement with the study by Gu *et al.* [26]. The present study found that the amounts of catechin and epicatechin in milk and white chocolates were below than 0.01 and 0.03 mg/mL, respectively. Results indicate epicatechin was predominant in dark chocolates. As reported by Cooper *et al*. [27], the ratio between epicatechin to catechin is 1 to 0.11. They also demonstrated that epicatechin content varies according to the manufacturing process due to epimerization of this compound to catechin. 

### Methylxanthine contents

The consumption of methylxanthines (caffeine, theobromine, and theophylline) and their potential physiological effects makes consumers desire to know the methylxanthine contents of foodstuffs. The present study found that theobromine could be found in dark and milk chocolates (Table 2). Theobromine levels were significantly higher in dark chocolates compared to milk chocolates, with 883.11 ± 3.54 and 125.54 ± 0.98 mg theobromine/100 g chocolate, respectively. The level of theobromine reported by Zoumas *et al.* [20] was similar with the results in the present study. However, Ramli *et al*. [24,25] found no significant difference in the methylxanthine levels between Malaysian commercial dark and milk chocolates. The amount of theobromine in white chocolate was below than 0.05 mg/mL (the LOD and LOQ for theobromine were 0.02 and 0.05 mg/mL, respectively). This was similar with the result obtained by Ministry of Agriculture, Fisheries, and Food (MAFF) [35], and may be due to the absence of added cocoa mass in white chocolate. 

**Table 2:** Theobromine in different types of commercial chocolates.

Type of chocolate	mg/100 g chocolate
Dark	883.11 ± 3.54
Milk	125.154 ± 0.98
White	BDL

Values are expressed as mean ± SD. Values are significant (p < 0.05) different between samples. The relative standard deviation was < 1%. BDL: Below detection limit.

Cocoa products are rich in caffeine and theobromine. Theobromine levels in chocolates was higher compared to other sources of methylxanthines such as carbonated beverages, coffee, and tea. However, methylxanthine-containing products such as chocolate chips, and milk chocolates, may not be accurately determined, as reported by Caudle *et al*. [36]. The present study showed that studied chocolates may contain little amounts of solid cocoa liquor/mass, which may lead to an underestimation of the methylxanthine content. According to Ministry of Agriculture, Fisheries, and Food (MAFF) [35], dark chocolates contained 237-519 mg of theobromine per 50 g portion. Michener and Rozin [37] reported that the main psychopharmacological active constituents of chocolates due to the present of high amount of methylxanthines in cocoa solid. The macronutrients (sugar and fat) did not significantly inhibit the uptake of methylxanthines in chocolates [19]. This indicated that, the effects of methylxanthines towards body systems remain the same even in dark, milk or white chocolates. Therefore, theobromine plays a significant role in reinforcing consumption of chocolates. They also reported that the psychopharmacological activity in chocolates to be confined to the combination of caffeine and theobromine. Since theobromine and caffeine always encounter together in cocoa products, thus, any behavioral effects following with cocoa consumption will depend on the quantity and bioavailability of methylxanthines [38].

## Conclusions

Both total phenolic and flavonoid contents in studied commercial chocolates were in the order of dark chocolates > milk chocolates > white chocolates. This could be due to the addition of different amount cocoa liquor or mass into the chocolates manufacturing. The highest phenolic content in dark chocolates compare to milk and white chocolates due to higher cocoa mass content. White chocolates with no addition of cocoa mass have possessed low phenolics and flavonoids contents. Apart from total phenolic content, theobromine was also detected in dark and milk chocolates. This study indicated that dark chocolates had contained higher phenolic antioxidant compounds compared to the other studied chocolates. 

## Experimental

### Sample collection and preparation

Three different types of chocolates namely dark, milk and white chocolate of various international and national brands were studied. For each type of chocolate a total of four national or international brands, representing commercial products available to customers, were randomly purchased from local markets in Kuala Lumpur, Malaysia. A composite sample was prepared from five samples of dark chocolates of the same brand, and the same preparation was also done for the milk and white chocolates. A total of 20 samples were analyzed for each chocolate type. The solid chocolates were chopped into small pieces and kept at -20 ºC before analysis.

### Chemicals

Catechin, sodium carbonate (Na_2_CO_3_), and sodium nitrite (NaNO_2_) were purchased from Sigma Aldrich (St Louis, MO, USA). Folin-Ciocalteu reagent, acetone, and *n*-hexane were purchased from Merck (Darmstadt, Germany). Sodium hydroxide (NaOH) was purchased from Hamburg Chemical (Heilbronn, Germany). Aluminum chloride (AlCl_3_) was purchased from BDH Chemical (Poole, England). Other common reagents used were of HPLC grade otherwise stated.

### Sample extraction

The samples extraction was according to the method used by Natsume *et al.* [16]. Samples of each type of chocolate (10 g) were triturated with *n*-hexane (3 x 50 mL) at room temperature for 30 min in order to remove most of the fats. The defatted chocolate (0.5 g) was extracted with 80% (v/v) acetone (3 x 50 mL) at 80 ºC, then the mixture was filtered through a filter paper (Whatman No. 1) using a Buchner funnel. This resulting solution was considered as polyphenol solution, which was used for polyphenols determination and HPLC analysis. 

### Determination of total phenolic content

The total phenolic content was determined according to the method of Lee *et al*. [34]. Briefly, appropriately diluted samples (1 mL) and a standard solution of catechin were transfer into a 25 mL volumetric flask containing distilled water (9 mL). A reagent blank using distilled water was also prepared. Folin-Ciocalteu phenol reagent (1 mL) was added to the mixture and been shaken. Following 5 min, 7% (w/v) Na_2_CO_3_ solution (10 mL) was added with mixing. The solution was then immediately diluted to a volume of 25 mL with distilled water and was mixed thoroughly. After incubation for 90 min at room temperature, the absorbance relative to that of a prepared blank at 750 nm was measured using a UV-Vis spectrophotometer (SECOMAM, Anthelie Advanced 5, France). The phenolic content was calculated based on the catechin calibration curve with the concentration of 0.2, 1, 5, 10, 15, and 20 μg/mL. The total phenolic contents of the samples were expressed as milligrams catechin equivalents (CAE) per serving size. 

### Determination of flavonoid content

The flavonoid content was measured using a colorimetric assay developed by Zhishen *et al*. [39]. Briefly, appropriately diluted sample (1 mL) was placed in a 10 ml volumetric flask containing distilled water (4 mL). At time zero, 5% NaNO_2_ (0.3 mL) was added to each volumetric flask; at 5 min, 10% AlCl_3_ (0.03 mL) was added; after 6 min, 1M NaOH (2 mL) was added. The mixture was then immediately diluted up to 10 mL with distilled water and was mixed thoroughly. The development of pink color was determined at 510 nm against blank. Catechin was used as the standard for a calibration curve. The flavonoid content was calculated using the linear equation based on the calibration curve with the concentration of 1, 5, 10, 15, and 20 μg/mL. The flavonoid of the samples was expressed as milligrams catechin equivalents (CAE) per serving size. 

### Analysis of individual polyphenols and methylxanthines by reversed-phase high performance liquid chromatography (HPLC)

The determination of individual polyphenols (catechin and epicatechin) and methylxanthine (theobromine) were done according to the method described by Natsume *et al*. [16]. Ten microlitres of each polyphenol solution was analyzed on HPLC with a C_18_ column (250 mm x 4.6 mm I.D., 5 µm), by using the solvents (A): 0.1% trifluoroacetic acid in acetonitrile (CH_3_CN) and (B): 0.1% trifluoroacetic acid in water. Elution was done with a linear gradient of 0 to 10% A in 5 min, 10 to 25% A in 25 min, and 25 to 100% A in 6 min (flow rate at 0.8 mL/min). Catechin and epicatechin were used as the standard for calibration curves. The catechin and epicatechin content were calculated using the linear equation based on the calibration curve with the concentration of 100, 200, 300, 400, and 500 μg/mL. The catechin and epicatechin contents of the samples were expressed as mg/100 g sample. Theobromine contents were calculated based on the calibration curve with the concentration of 200, 250, 400 and 600 μg/mL. The theobromine contents of the samples were expressed as mg/100 g sample. 
